# Facial expressions contribute more than body movements to conversational outcomes in avatar-mediated virtual environments

**DOI:** 10.1038/s41598-020-76672-4

**Published:** 2020-11-26

**Authors:** Catherine Oh Kruzic, David Kruzic, Fernanda Herrera, Jeremy Bailenson

**Affiliations:** Virtual Human Interaction Lab, Department of Communication, Stanford University, 450 Serra Mall, Stanford, CA 94305 USA

**Keywords:** Psychology, Human behaviour

## Abstract

This study focuses on the individual and joint contributions of two nonverbal channels (i.e., face and upper body) in avatar mediated-virtual environments. 140 dyads were randomly assigned to communicate with each other via platforms that differentially activated or deactivated facial and bodily nonverbal cues. The availability of facial expressions had a positive effect on interpersonal outcomes. More specifically, dyads that were able to see their partner’s facial movements mapped onto their avatars liked each other more, formed more accurate impressions about their partners, and described their interaction experiences more positively compared to those unable to see facial movements. However, the latter was only true when their partner’s bodily gestures were also available and not when only facial movements were available. Dyads showed greater nonverbal synchrony when they could see their partner’s bodily and facial movements. This study also employed machine learning to explore whether nonverbal cues could predict interpersonal attraction. These classifiers predicted high and low interpersonal attraction at an accuracy rate of 65%. These findings highlight the relative significance of facial cues compared to bodily cues on interpersonal outcomes in virtual environments and lend insight into the potential of automatically tracked nonverbal cues to predict interpersonal attitudes.

## Introduction

Nonverbal cues are often heralded as the main source of social information during conversations. Despite the many decades social scientists have studied gestures, however, there are only a handful of large sample studies in which the body movements of interactants are measured in detail over time and associated with various communication outcomes. Hence, this experiment capitalizes on dramatic advancements in virtual reality (VR) technology to track and quantify the facial expressions and body movements of over 200 people speaking to one another while embodied in an avatar.

Steuer^1^ defines VR as “a real or simulated environment in which a perceiver experiences telepresence.” Under this definition, VR includes immersive and non-immersive experiences involving technologies that contribute to feelings of vividness and interactivity, the two core dimensions of telepresence^1^^.^ Multiple companies have launched avatar-mediated social VR platforms, which allow users to connect with others using customized avatars (i.e., digital representations of users controlled in real-time^2^) in virtual environments. One development that has made avatar-mediated communication particularly attractive has been the possibility to achieve unprecedented levels of behavioral realism^3^. Optical tracking systems (e.g., HTC Vive, Microsoft Kinect, Oculus Rift CV1) can measure users’ physical movements in real-time with great accuracy^4^ and render virtual representations accordingly. Although less common in consumer products, developments in computer vision allow for facial tracking through information extracted from RGB and/or infrared cameras. While facial tracking is yet to be widely available on social VR platforms, there has been a growing interest in developing technology that allows for a more seamless facial tracking experience^5–7^.

Despite the significant interest in adding nonverbal cues to VR, little is known about the impact of incorporating nonverbal channels in avatar-mediated environments. While current industrial trends appear to revolve around the belief that ‘more is better’, studies show that technical sophistication does not necessarily lead to more favorable outcomes^8,9^ Furthermore, considering that even minimal social cues are enough to elicit social responses^10^ and that verbal strategies are sufficient to communicate emotional valence^11^, it is unclear whether incorporating additional nonverbal cues will linearly improve communication outcomes.

Understanding the impact of facial expressions and bodily movements within avatar-mediated environments can help further our understanding of the significance of these channels in FtF contexts. While there are a handful studies that lend insight into the independent and joint contributions of various nonverbal channels during FtF interactions, the majority of these studies were either conducted with static images^12,13^ or posed expressions^14–16^, rather than FtF interactions. In addition, the limited number of studies that did study the impact of different nonverbal cues in FtF dyadic contexts asked participants to wear sunglasses^17,18^ or covered parts of their bodies^19,20^, which inevitably alters the appearance of the target individual and reduces both the ecological validity and generalizability of results. By using identical avatars across conditions and only allowing the nonverbal information to differ, the present study offers an ideal balance between experimental control and ecological validity^3^.

## Behavioral realism and interpersonal outcomes

The extant literature offers a mixed picture regarding the relationship between nonverbal cues and interpersonal outcomes within avatar-mediated contexts. On the one hand, studies show that increasing behavioral realism can improve communication outcomes^21,22^. Moreover, past studies have demonstrated that increasing behavioral realism by augmenting social cues exhibited by avatars (e.g., eye gaze and facial expressions) can enhance collaboration and produce meaningful interactions^23–25^. It is important to note, however, that the nonverbal cues included in these studies often manipulated responsive behaviors (e.g., mutual gaze, nodding), which are associated with positive outcomes^26,27^. As such, it is uncertain if the purported benefits of behavioral realism were due to the addition of nonverbal cues or perceptions of favorable nonverbal behavior.

In contrast, other studies^28,29^ found that general levels of behavioral realism do not uniformly improve communication outcomes. For instance, two studies^30,31^ found that adding facial expressions or bodily gestures to avatar-mediated virtual environments did not consistently enhance social presence or interpersonal attraction. However, both of these studies employed a task-oriented interaction without time limits and a casual social interaction, which may have given participants enough time and relevant social information to reach a ceiling effect regardless of the nonverbal cues available. This is a reasonable conjecture, considering that increased interaction time can allow interactants to overcome the lack of nonverbal cues available in CMC^32^. As such, the effects of nonverbal cues independent of increased time or availability of social content are unclear. In addition, despite ample research that points to the association between interpersonal judgments based on nonverbal behavior^33^, most studies did not utilize the automatically tracked nonverbal data to explore its association with interpersonal outcomes which could further our understanding of the sociopsychological implications of automatically tracked nonverbal cues.

Taking these limitations into account, the present study attempts to elucidate the unique influences of including facial expressions and bodily gestures on interaction outcomes (i.e., interpersonal attraction, social presence, affective valence, impression accuracy) by employing a goal-oriented task with time constraints. The present study also offers a less constricted representation of participants’ nonverbal behavior including expressions of negative and/or neutral states, rather than limiting the available nonverbal cues related to feedback or friendliness (e.g., head nodding, reciprocity, smiling).

## Predicting interpersonal attraction with automatically detected nonverbal cues

Nonverbal cues not only influence impression formation, but also reflect one’s attitude toward their communication partner(s)^34,35^ such as interpersonal attraction^31^, bonding^36^, and biased attitudes^37^. In addition to nonverbal cues that are isolated to the individual, studies have shown that interactional synchrony is associated with more positive interpersonal outcomes^38–41^. Interactional synchrony is defined as the “the temporal linkage of nonverbal behavior of two or more interacting individuals”^42^. Under this definition, synchrony refers to the motion interdependence of all participants during an interaction focusing on more than a single behavior (e.g., posture or eye gaze). This view of synchrony is consistent with Ramseyer and Tschacher’s^39^ characterization of synchrony and is grounded within the dynamical systems framework^43^. Interactional synchrony has been associated with the ability to infer the mental states of others^44^ and rapport^45^. For example, spontaneous synchrony was related to Theory of Mind^46^ for participants with and without autism, such that increased synchrony was associated with higher ability to infer the feelings of others^47^.

While research has consistently found that nonverbal behavior is indicative of interpersonal outcomes^38^, the vast majority of these studies quantified nonverbal behavior by using human coders who watched video recordings of an interaction and recorded the target nonverbal behaviors or Motion Energy Analysis (MEA; automatic and continuous monitoring of the movement occurring in pre-defined regions of a video). Coding nonverbal behavior by hand is not only slow and vulnerable to biases^42,48^, but also makes it difficult to capture subtle nonverbal cues that aren’t easily detectible by the human eye. While MEA is more efficient than manual coding, it is limited in that it is based on a frame-by-frame analysis of regions of interest (ROI) and thus susceptible to region-crossing (i.e., movement from one region being confused with that of another region^49^). That is, MEA does not track individual parts of the body, but pixels within ROI. Given these limitations, researchers have recently turned to the possibility automating the quantification of nonverbal behavior by capitalizing upon dramatic improvements in motion detection technology (e.g., tracking with RGB-D cameras) and computational power (e.g., machine learning)^36,42,50^. While these methods are also prone to tracking errors, they have the advantage of tracking nonverbal cues in a more targeted manner (i.e., specific joints, facial expressions) and offer higher precision by utilizing depth data in addition to color (RGB) data.

While researchers have started to employ machine learning algorithms to determine the feasibility of using automatically detected nonverbal cues to predict interpersonal outcomes, they either relied solely on isolated nonverbal behaviors^36^ or entirely on nonverbal synchrony^42,51^ instead of both isolated and interdependent nonverbal cues. In addition, previous studies have employed relatively small sample sizes (N_*dyad*_ range: 15–53). Perhaps for this reason, prior machine learning classifiers either performed above chance level only when dataset selection was exclusive^42,51^ or showed unreliable performance in terms of validation and testing set accuracy rates^36^. Consequently, there is inconclusive evidence if automatically tracked nonverbal cues can reliably predict interpersonal attitudes. By employing machine learning algorithms to explore whether nonverbal behaviors can predict interpersonal attitudes, the present study aims to address if and, if so how, automatically tracked nonverbal cues and synchrony are associated with interpersonal outcomes through an inductive process.

## Methods

### Study design

**Figure 1:**
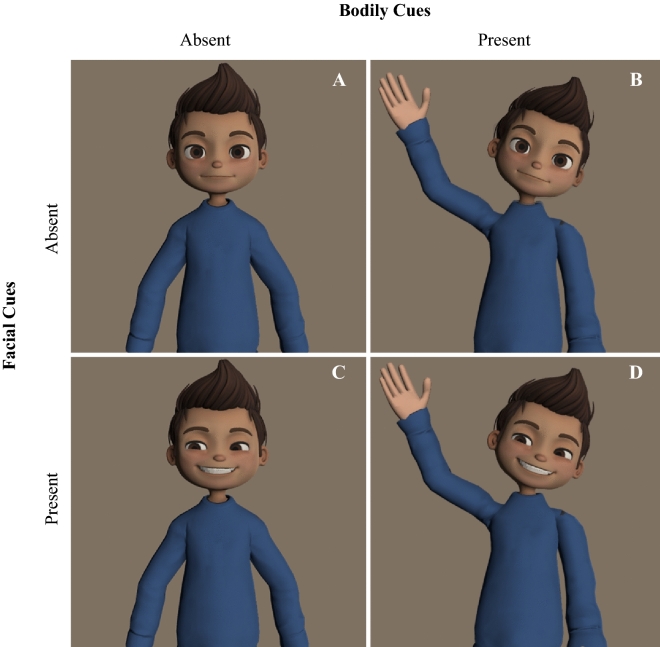
Graphical representations of the four conditions: static avatar (**A**), body only (**B**), face only (**C**), body and face (**D**).

The present study adopted a 2 Bodily Gestures (Present vs. Absent) × 2 Facial Expressions (Present vs. Absent) between-dyads design. Dyads were randomly assigned to one of the four conditions, and gender was held constant within a dyad. There was an equal number of male and female dyads within each condition. Participants only interacted with each other via their avatars and did not meet or communicate directly with each other prior to the study. The nonverbal channels that were rendered on the avatar were contingent on the experimental condition. Participants in the ‘Face and Body’ condition interacted with an avatar that veridically portrayed their partner’s bodily and facial movements. Participants in the ‘Body Only’ condition interacted with an avatar that veridically represented their partner’s bodily movements, but did not display any facial movements (i.e., static face). In contrast, participants in the ‘Face Only’ condition interacted with an avatar that veridically portrayed their partner’s facial movements, but did not display any bodily movements (i.e., static body). Finally, participants in the ‘Static Avatar’ condition interacted with an avatar that did not display any movements. A graphical representation of each condition is available in Fig. 1.

### Participants

Participants were recruited from two medium-sized Western universities (Foothill College, Stanford University). Participants were either granted course credit or a $40 Amazon gift card for their participation. 280 participants (140 dyads) completed the study. Dyads that included participants who failed the manipulation check (*N*_*dyad*_ = 10) and/or participants who recognized their partners (*N*_*dyad*_ = 6) were excluded from the final analysis. To determine if participants who were part of a specific condition were more likely to fail the manipulation check or to recognize their interaction partners, two chi-square tests were conducted. Results indicated that there were no differences between conditions for either dimension (manipulation check failure: *χ*^*2*^(3) = 1.57, *p* = 0.67, partner recognition: *χ*^*2*^(3) = 1.78, *p* = 0.62).

### Materials and apparatus

**Figure 2:**
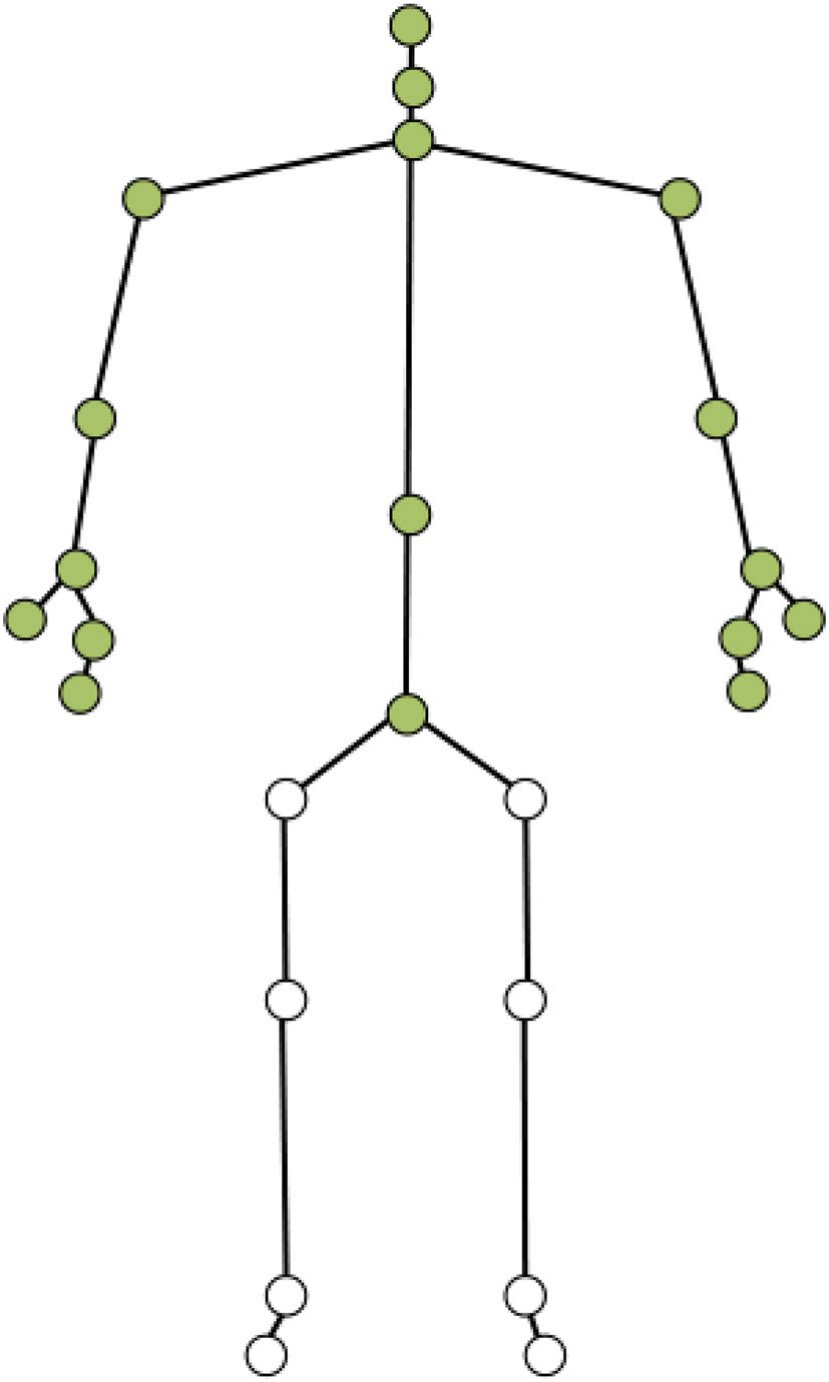
Joints tracked by the kinect: only colored joints were mapped to avatar.

A markerless tracking device (Microsoft Kinect for Xbox One with adaptor for Windows) was used to track participants’ bodily gestures. Using an infrared emitter and sensor, the Microsoft Kinect is able to provide the positional data for 25 skeletal joints at 30 Hz in real-time, allowing unobtrusive data collection of nonverbal behavior. Studies offer evidence that the Kinect offers robust and accurate estimates of bodily movements^52^. While even higher levels of accuracy can be achieved with marker-based systems, this study employed a markerless system to encourage more naturalistic movements^53^. The joints that are tracked by the Kinect are depicted in Fig. 2. The present study used 17 joints that belong to the upper body as studies have suggested that the Kinect tends to show poorer performance for lower body joints^52^ (i.e., left hip, right hip, left knee, right knee, left ankle, right ankle, left foot, right foot), which can result in “substantial systematic errors in magnitude” of movement^54^.

Participants’ facial expressions were tracked in real-time using the TrueDepth camera on Apple’s iPhone XS. The TrueDepth camera creates a depth map and infrared image of the user’s face, which represents the user’s facial geometry^55^. More specifically, the TrueDepth camera captures an infrared image of the user’s face and projects and analyzes approximately 30,000 points to create a depth map of the user’s face, which are subsequently analyzed by Apple’s neural network algorithm. Among other parameters, Apple’s ARKit SDK can extract the presence of facial expressions from the user’s facial movements. A full list of the 52 facial expressions that are tracked by ARKit are included in “Appendix 1”. The value of the facial expression (i.e., blendshape) ranges from 0 to 1 and is determined by the current position of a specific facial movement relative to its neutral position^55^. Each blendshape was mapped directly from the participant’s facial movements. While we do not have a quantitative measure for tracking accuracy, qualitative feedback from pilot sessions with 40 participants suggested that participants found the facial tracking to be accurate.

**Figure 3:**
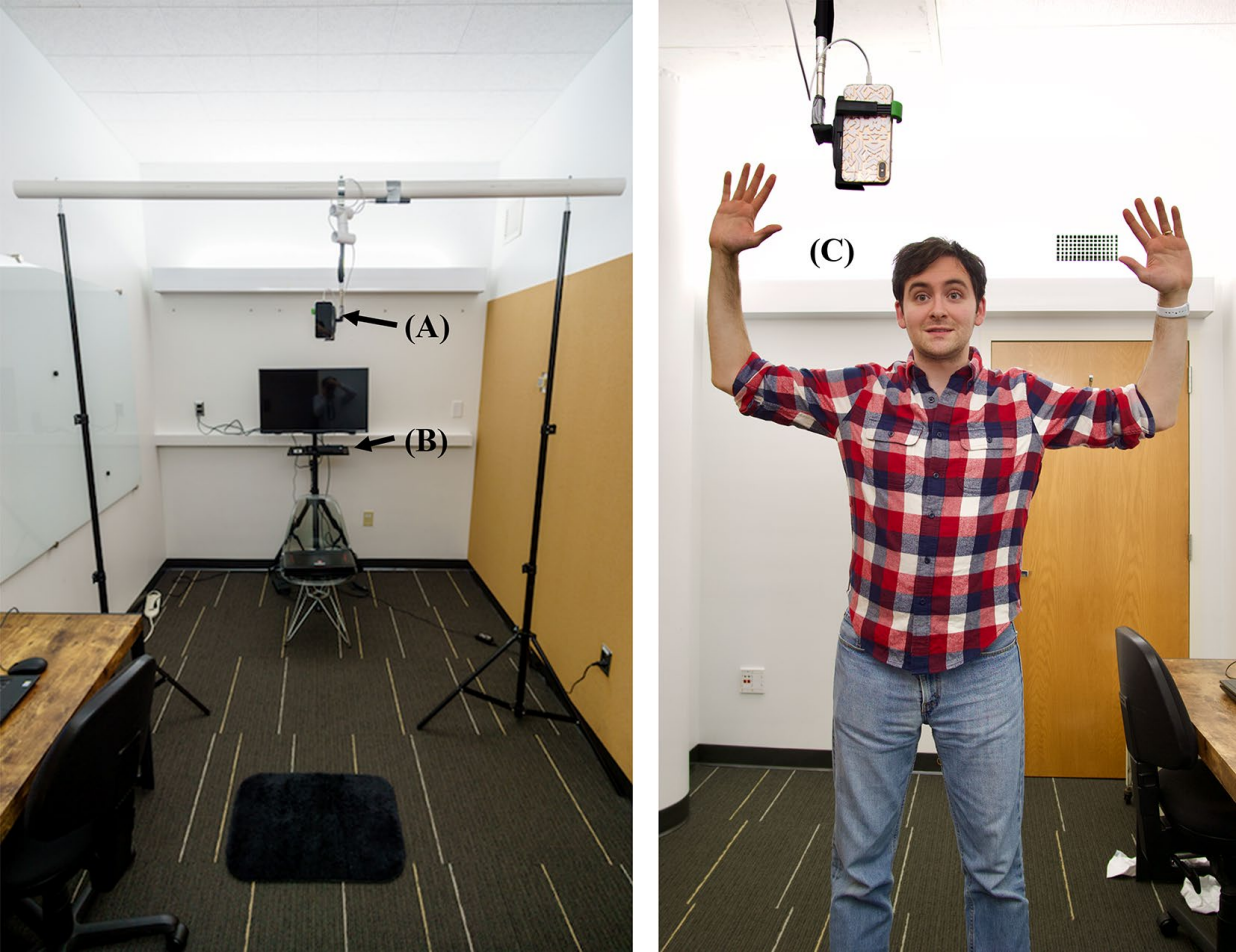
Configuration of study room (left): (**A**) iPhone XS for facial tracking, (**B**) Kinect for Xbox One for body tracking, (**C**) person being tracked during visual referential task.

Discord, one of the most commonly used Voice over Internet Protocol (VoIP) platforms^56^, was used for verbal communication. Participants were able to hear their partner’s voice through two speakers (Logitech S120 Speaker System) and their voices were detected by the microphone embedded in the Kinect sensor. Participants were able to see each other’s avatars on a television (Sceptre 32" Class FHD (1080P) LED TV (X325BV-FSR)), which was mounted on a tripod stand (Elitech). The physical configuration of the study room can be seen in Fig. 3. The person pictured in Fig. 3 gave informed consent to publish this image in an online open-access publication. The avatar-mediated platform in which participants interacted was programmed using Unity version 2018.2.2. Further details of the technical setup are available in “Appendix 2” and information regarding the system’s latency can be seen in “Appendix 3”.

### Procedure

All study procedures and materials received approval from the Institutional Review Board of Stanford Univeristy. All methods were performed in accordance with relevant guidelines and regulations. Participants in each dyad were asked to come to two separate locations to prevent them from seeing and interacting with each other prior to the study. Participants were randomly assigned to one of the two study rooms, which were configured identically (Fig. 3). Once participants gave informed consent to participate in the study, they completed a pre-questionnaire that measured their personality across five dimensions^57^ (extraversion, agreeableness, neuroticism, conscientiousness, openness to experience). After each participant completed the pre-questionnaire the experimenter explained that two markerless tracking systems would be used to enable the participant and their partner to interact through the avatar-mediated platform. The participant was then asked to stand on a mat measuring 61 cm × 43 cm that was placed 205 cm away from the Kinect and 20 cm away from the iPhone XS. After the participant stood on the mat, the experimenter asked the participant to confirm that the phone was not obstructing her/his view. If the participant said that the phone was blocking his/her view, the height of the phone was adjusted. Upon confirming that the participant was comfortable with the physical setup of the room and that the tracking systems were tracking the participant, the experimenter opened the avatar-mediated platform and let the participants know that they would be completing two interaction tasks with a partner. After answering any questions that the participants had, the experimenter left the room.

**Figure 4:**
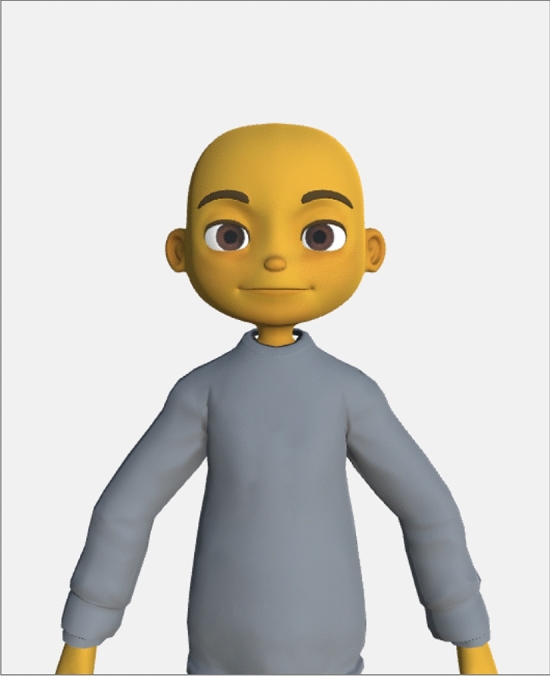
Avatar used during calibration phase.

Prior to the actual interaction, participants went through a calibration phase. During this time, participants were told that they would be completing a few calibration exercises to understand the physical capabilities of the avatars. This phase helped participants familiarize themselves to the avatar-mediated platform and allowed the experimenter to verify that the tracking system was properly sending data to the avatar-mediated platform. Specifically, participants saw a ‘calibration avatar’ (Fig. 4) and were asked to perform facial and bodily movements (e.g., raise hands, tilt head, smile, frown). The range of movement that was visualized through the calibration avatar was consistent with the experimental condition of the actual study. All participants were asked to do the calibration exercises regardless of condition in order to prevent differential priming effects stemming from these exercises and demonstrate the range of movements that could be expected from their partner’s avatars.

After completing the calibration exercises, participants proceeded to the actual study. Participants were informed that they would collaborate with each other to complete two referential tasks: an image-based task (i.e., visual referential task) and a word-based task (i.e., semantic referential task). The order in which the tasks were presented was counterbalanced across all conditions.

**Figure 5:**
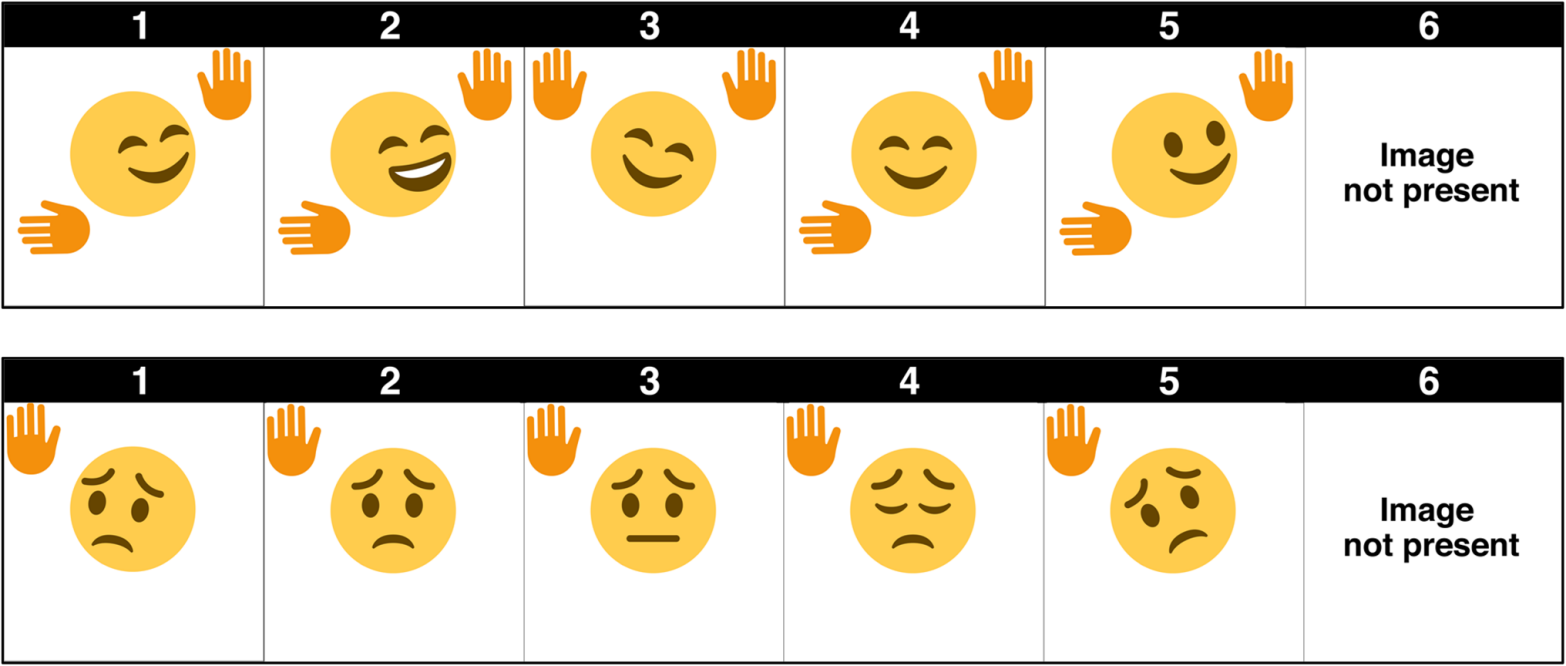
Examples of stimuli for visual referential task.

The image-based task was a figure-matching task adapted from Hancock and Dunham^58^. Each participant was randomly assigned the role of the ‘Director’ or the ‘Matcher’. The Director was asked to describe a series of images using both verbal and nonverbal language (e.g., tone/pitch of voice, body language, facial expressions). The Matcher was asked to identify the image that was being described from an array of 5 choices and one “image not present” choice and to notify the Director once he or she believed the correct image had been identified (Fig. 5). Both the Matcher and Director were encouraged to ask and answer questions during this process. The Matcher was asked to select the image that he or she believed was a match for the image that the Director was describing; if the image was not present, the Matcher was asked to select the “image not present” choice. After 7 min or after participants had completed the entire image task (whichever came first), participants switched roles and completed the same task one more time.

The word-based task was a word-guessing task adapted from the ‘password game’ used in Honeycutt, Knapp, and Powers^59^^.^ Each participant was randomly assigned the role of the ‘Clue-giver’ or the ‘Guesser’. The Clue-giver was asked to give clues about a series of thirty words using both verbal and nonverbal language. The Guesser was asked to guess the word that was being described. Both the Clue-giver and the Guesser were encouraged to ask and answer questions during this process. Given the open-ended nature of the task, participants were told that they were allowed to skip words if they thought that the word was too challenging to describe or guess. After 7 min or after they had completed the word task (whichever came first), participants switched roles and completed the same task one more time; the Clue-giver became the Guesser and the Guesser became the Clue-giver. The words used in the word-based task were chosen from *A Frequency Dictionary of Contemporary American English*^60^, which provides a list of 5,000 of the most frequently used words in the US; 90 words were chosen from the high, medium, and low usage nouns and verbs from this list. The selected words were presented in a random order for the Clue-giver to describe.

These tasks were chosen for the following reasons: first, two types of referential tasks (i.e., visual and semantic) were employed in order to reduce the bias of the task itself toward verbal or nonverbal communication. That is, the visual task was selected as a task more amenable to nonverbal communication, while the semantic task was selected as one more amenable to verbal communication. Second, we adopted a task-oriented social interaction to avoid ceiling effects of the interpersonal outcome measures, given that purely social exchanges are more likely to support personal self-disclosures, which are associated with interpersonal attraction and facilitate impression formation.

After the interaction, participants completed the post-questionnaire which assessed perceptions of interpersonal attraction, affective valence, impression accuracy, and social presence. Participants’ bodily and facial nonverbal data were tracked and recorded unobtrusively during the interaction. As noted in “Methods”, participants gave consent for their nonverbal data to be recorded for research purposes. Once they completed the post-questionnaire, participants were debriefed and thanked.

### Measures

#### Interpersonal attraction

Based on McCroskey and McCain^61^, two facets of interpersonal attraction were measured, namely social attraction and task attraction. Social attraction was measured by modifying four items from Davis and Perkowitz^62^ to fit the current context and task attraction was assessed by modifying four items from Burgoon^63^. Participants rated how strongly they agreed or disagreed with each statement on a 7 point Likert-type scale (1 = *Strongly Disagree*, 7 = *Strongly Agree*). The wording for all questionnaire measures is included in “Appendix 4”.

**Figure 6:**
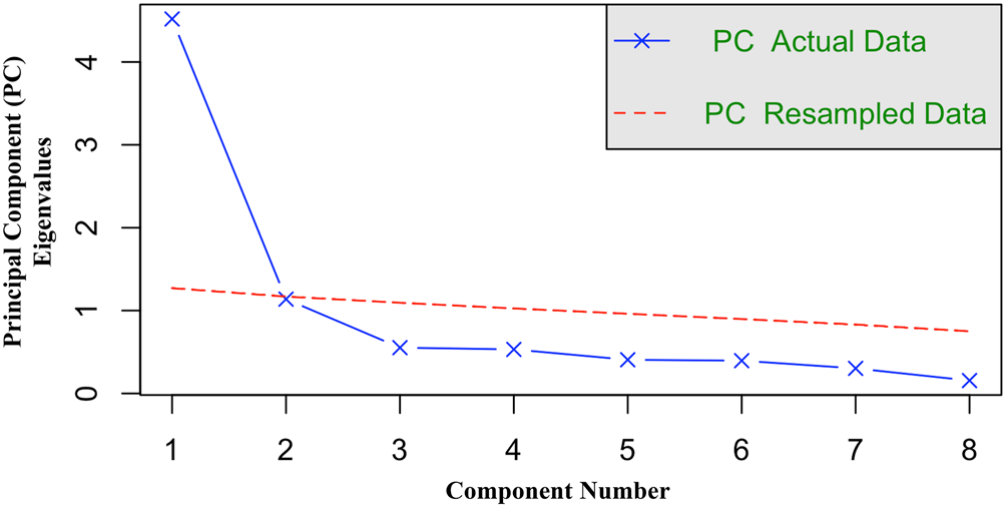
Parallel analysis scree plots of actual and resampled interpersonal attraction data.

Due to the similarity of the social and task attraction scales, a parallel analysis^64^ (PA) was run to determine the correct number of components to extract from the eight items. PA results indicated that the data loaded on to a single component, as indicated by Fig. 6. A confirmatory factor analysis with varimax rotation showed that 56% of the variance was explained by the single component, and that the standardized loadings for all items were greater than 0.65 (Table 1). Thus, the two subscales of interpersonal attraction were collapsed into a single measure of interpersonal attraction. The reliability of the scale was good, Cronbach’s *α* = 0.89. Greater values indicated higher levels of interpersonal attraction (*M* = 5.84, *SD* = 0.61); the minimum was 3.75 and the maximum was 7.

**Table 1:** Factor analysis of interpersonal attraction with varimax rotation.

Item #	Question	Factor 1
1	I enjoyed completing the tasks with my partner	.80
2	I had fun completing the tasks with my partner	.78
3	I would like to interact with my partner again	.79
4	It was interesting to complete the tasks with my partner	.70
5	I like my partner	.76
6	I would get along well with my partner	.77
7	I would enjoy a casual conversation with my partner	.72
8	My partner is friendly	.68
Eigenvalue		4.52
% of variance explained		56%
Cronbach’s *α*		.89

#### Affective valence

A Linguistic Inquiry Word Count^65^ (LIWC) analysis was performed on an open-ended question that asked participants to describe their communication experience. LIWC has been used as a reliable measure for various interpersonal outcomes, including the prediction of deception^66^, personality^67^, and emotions^68^. Affective valence was computed by subtracting the percentage of negative emotion words from the percentage of positive emotion words yielded by the LIWC analysis^69^. Greater values indicated relatively more positive affect than negative affect (*M* = 3.59, *SD* = 3.4); the minimum was − 2.94 and the maximum was 20.

#### Impression accuracy

Participants completed a self and an observer version of the short 15-item Big Five Inventory^70,71^ (BFI-S). Participants rated themselves and their partner on 15 items that were associated with five personality dimensions (i.e., extraversion, agreeableness, conscientiousness, neuroticism, and openness to experience) on a 7 point Likert-type scale (1 = *Strongly Disagree*, 7 = *Strongly Agree*). Participants were given the option to select “Cannot make judgment” for the observer version of the BFI-S.

Impression accuracy was defined as the profile correlation score, which “allows for an examination of judgments in regard to a target's overall personality by the use of the entire set of […] items in a single analysis”^72^; that is, impression accuracy was assessed by computing the correlation coefficient across the answers that each participant and their partner gave for the 15 items^72,73^. Greater values indicated more accurate impressions (*M* = 0.39, *SD* = 0.36); the minimum was − 0.64 and the maximum was 0.98.

#### Social presence

Social presence was measured with items selected from the Networked Minds Measure of Social Presence^74,75^, one of the most frequently used scales to measure social presence. To reduce cognitive load, 8 items were selected from the scale, which consisted of statements that assessed co-presence, attentional engagement, emotional contagion, and perceived comprehension during the virtual interaction. Participants rated how strongly they agreed or disagreed with each statement on a 7 point Likert-type scale (1 = *Strongly Disagree*, 7 = *Strongly Agree*). The reliability of the scale was good, Cronbach’s *α* = 0.77. Greater values indicated higher levels of social presence (*M* = 5.47, *SD* = 0.65); the minimum was 3.38 and the maximum was 6.75.

#### Nonverbal behavior

Participants’ bodily movements were tracked with the Microsoft Kinect. Due to non-uniform time distances in the tracking data, linear interpolation was used to interpolate the data to uniform time distances of 30 Hz. Then, a second-order, zero-phase bidirectional, Butterworth low-pass filter was applied with a cutoff frequency of 6 Hz to provide smooth estimates^76^. Participants’ facial expressions were tracked in real-time using the TrueDepth camera on Apple’s iPhone XS and this data was also interpolated to 30 Hz.

#### Synchrony of bodily movement

Synchrony of bodily movements is defined as the correlation between the extent of bodily movements of the two participants, with higher correlation scores indicating higher synchrony. More specifically, the time series of the extent of bodily movements of the two participants were cross-correlated for 100 s of the interaction. Cross-correlation scores were computed for both positive and negative time lags of five seconds, in accordance to Ramseyer and Tschacher^39^, which accounted for both ‘pacing’ and ‘leading’ synchrony behavior. Time lags were incremented at 0.1 s intervals, and cross-correlations were computed for each interval by stepwise shifting one time series in relation to the other^39^. While the Kinect can capture frames at 30 Hz, the sampling rate varies and the resulting data is noisy. During post-processing, we addressed both shortcomings by filtering and downsampling to a uniform frequency. As noted above, a Butterworth low-pass filter with a cutoff frequency of 6 Hz was applied to remove signal noise, and then was interpolated to 10 Hz to achieve a uniform sampling rate across the body and face. In instances wherein less than 90% of the data were tracked within a 100 s interval, the data from that interval were discarded. Participants’ synchrony scores were computed by averaging the cross-correlation values.

#### Synchrony of facial expressions

**Figure 7:**
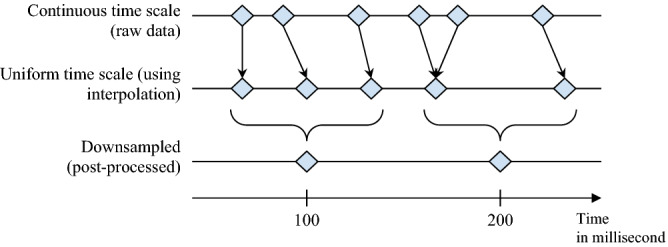
Illustration of post-processing sequence for facial movement data.

Synchrony of facial expressions is similarly defined as the correlation between the time series of facial movements. Once again, the time series of facial movements of the two participants were cross-correlated for each 100 s interval of the interaction. Cross-correlations were computed for both positive and negative time lags of 1 s, in accordance with Jaques et al.^36^). Time lags were incremented at 0.1 s intervals, and cross-correlations were computed for each interval by stepwise shifting one time series in relation to the other. The facial tracking data was downsampled to 10 Hz to compensate for gaps that were introduced after the data was mapped from a continuous to a uniformly spaced time scale. (Fig. 7). Once again, if less than 90% of the data were tracked within a given 100 s interval, the data from that interval were discarded. Participants’ synchrony scores were computed by averaging the cross-correlation values.

#### Extent of bodily movement

To assess the extent to which participants moved their body, the between-second Euclidean distance for each joint was computed across the interaction. This is equivalent to the Euclidean distance for each joint for each 0.03 s (30 Hz). The average Euclidian distance for each 0.03 s interval for each joint was then averaged across the 17 joints to form a single composite score.

#### Extent of facial movement

To assess the extent of facial movement during the interaction, the confidence scores for each facial movement (i.e., the deviation of each facial movement from the neutral point) was sampled at a rate of 30 Hz and averaged to form a single composite score. Facial expressions that had a left and right component (e.g., Smile Left and Smile Right) were averaged to form a single item. Finally, facial movements that showed low variance during the interaction were excluded to avoid significant findings due to spurious tracking values.

#### Machine learning

Machine learning is defined “a set of methods that can automatically detect patterns in data, and then use the uncovered patterns to predict future data, or to perform other kinds of decision making under uncertainty”^77^. Machine learning is an inductive method which can be used to process large quantities of data to produce bottom-up algorithms^42^. This makes machine learning suitable for discovering potential patterns within millions of quantitative nonverbal data points. Two machine learning algorithms—random forest and a neural network model (multilayer perceptron; MLP)—that used the movement data as the input layer and interpersonal attraction as the output layer were constructed. To allow for the machine learning algorithm to function as a classifier, participants were divided into high and low interpersonal attraction groups based on a median split^78^. Then, the dataset was randomly partitioned into a training (70%) and test dataset (30%).

There were 827 candidate features for the input layer; bodily synchrony among 17 joints and 10 joint angles^42^; facial synchrony among the 52 facial expressions (“Appendix 1”; four different types of nonverbal synchrony were included as candidates: mean cross-correlation score, absolute mean of cross-correlation scores, mean of non-negative cross-correlation scores, and maximum cross-correlation score); the mean, standard deviation, mean of the gradient, standard deviation of the gradient, maximum of the gradient, and maximum of the second gradient for each joint coordinate (i.e., X, Y, Z); the mean and standard deviation of the Euclidean distance for each joint for each 0.1 s interval; the mean, standard deviation, mean of the absolute of the gradient, and the standard deviation of the absolute of the gradient for the joint angles; the mean and standard deviations of the head rotation (i.e., pitch, yaw, roll); the mean and standard deviations of the gradient of the head rotation; the mean and standard deviations of the 52 facial expressions; the mean and standard deviation of the X and Y coordinates of point of gaze; the percentage of valid data and the number of consecutive missing data points; gender.

Two methods of feature selection were explored for the training set. First, features were selected using a correlation-based feature selection method, wherein features that highly correlated with the outcome variable, but not with each other were selected^79^. Then, support vector machine recursive feature elimination^80^ was used to reduce the number of features and identify those that offered the most explanatory power. The test dataset was not included in the data used for feature selection. 23 features were selected using this method (Table 2).

**Table 2:** Features selected.

Channel	Nonverbal cue	Input feature
Face	Upward compression of lower left lip	Absolute maximum of cross-correlation score
		Mean of cross-correlation score
	Upward compression of lower right lip	Absolute maximum of cross-correlation score
		Mean of cross-correlation score
	Upward movement of left mouth corner	Absolute maximum of cross-correlation score
		Mean extent of movement
	Outward movement of upper lip	Mean extent of movement
	Upward gaze of right eye	Absolute mean of cross-correlation score
	Inward gaze of left eye	Mean extent of movement
Body	Head joint	Mean of y position
	Neck joint	Absolute maximum of cross-correlation score
	Spine shoulder joint	Absolute maximum of cross-correlation score
	Right elbow angle	Absolute maximum of cross-correlation score
		Maximum of the gradient
		Maximum of the second gradient
		Mean of x position
	Right hand tip joint	Maximum of the gradient
		Maximum of the second gradient
	Right shoulder joint	Mean of x position
	Left wrist joint	Absolute maximum of cross-correlation score
		Mean of x position
	Left hand angle	Absolute maximum of cross-correlation score
	Left shoulder angle	Standard deviation

Using five-fold cross-validation, the selected features were used to train two different machine learning models (i.e., random forest, MLP) in order to assess initial model performance. More specifically, five-fold cross-validation was used to validate and tune the model performance given the training dataset prior to applying the classifier to the holdout test data. Five-fold cross-validation divides the training set into five samples that are roughly equal in size. Among these samples, one is held out as a validation dataset, while the remaining samples are used for training; this process is repeated five times to form a composite validation accuracy score (i.e., the percentage of correctly predicted outcomes).

### Statistical analyses

Data from participants who communicate with each other are vulnerable to violating the assumption of independence and are thus less appropriate for ANOVA and standard regression approaches^81^. Multilevel analysis “combines the effects of variables at different levels into a single model, while accounting for the interdependence among observations within higher-level units”^82^. Because neglecting intragroup dependence can bias statistical estimates including error variance, effect sizes and *p* values^83,84^, a multilevel model was used to analyze the data. Random effects that arise from the individual subjects who are nested within dyads were accounted for and a compound symmetry structure was used for the within-group correlation structure. Gender was included as a control variable, as previous research has found that females tend to report higher levels of social presence than their male counterparts^85^. In line with these studies, correlation analyses (Table 3) showed that gender correlated with several of the dependent variables. A summary of the results of the multilevel analyses are available in Table 4.

**Table 3:** Bivariate Pearson correlations of variables.

Measures	1	2	3	4	5	6	7	8	9
1. Social presence									
2. Interpersonal attraction	.57***								
3. Affective valence	.24**	.20**							
4. Impression accuracy	.16*	.18**	.14*						
5. Bodily movement (extent)	.18*	.16*	.11^†^	− .09					
6. Facial movement (extent)	.18**	.21***	.20**	.08	.21**				
7. Bodily synchrony	.12^†^	.09	− .06	.01	− .02	.14*			
8. Facial synchrony	.24***	.30***	.09	.03	− .02	.09	.40***		
9. Gender	.22***	.22***	.06	.00	.13^†^	0.07	.08	.39***	
10. Task order	− .01	.00	.08	− .05	.01	.01	− .10^†^	− .14*	.02

^†^*p* < .10, *p < .05, **p < .01, ***p < .001.

**Table 4:** Summary of multilevel analyses.

	Interpersonal attraction	Affective valence	Impression accuracy	Social presence	Bodily movement	Facial movement	Bodily synchrony	Facial synchrony
	*B* (*SE*)	*B* (*SE*)	*B* (*SE*)	*B* (*SE*)	*B* (*SE*)	*B* (*SE*)	*B* (*SE*)	*B* (*SE*)
Body	.09* (.04)	.39 (.21)	.06* (.02)	.04 (.04)	.02*** (.01)	.001 (.001)	.002^†^ (.001)	.01*** (.003)
Face	− .02 (.04)	− .16^†^ (.21)	− .01 (.02)	.04 (.04)	− .01 (.01)	− .0004 (.001)	.002 (.001)	− .0002 (.003)
Gender	.28*** (.08)	.45 (.46)	.001 (.05)	.29*** (.08)	.01 (.01)	.003 (.003)	.002 (.002)	.02*** (.005)
Body * Face	.05 (.04)	.46* (.21)	.03 (.02)	.06 (.04)	.01* (.01)	.002 (.001)	− .001 (.001)	.00004 (.003)
AIC	470.97	1320.62	217.46	507.25	− 480.53	− 1164.98	− 1404.92	− 1331.03
BIC	498.91	1348.57	245.13	535.19	− 452.72	− 1137.10	− 1377.04	− 1303.18

^†^*p* < .10, *p < .05, **p < .01, ***p < .001.

## Results

### Manipulation check

To confirm that the manipulation of the nonverbal variables was successful, participants were asked if the following two sentences accurately described their experience (0 = *No*, 1 = *Yes*): “My partner's avatar showed changes in his/her facial expressions, such as eye and mouth movements” and “My partner's avatar showed changes in his/her bodily gestures, such as head and arm movements”. 11 participants who belonged to 10 separate dyads failed the manipulation check; these participants and their partners were removed from the final data analyses (*N*_*dyad*_ = 10, *N*_*participant*_ = 20).

An additional 7 participants who belonged to 6 separate dyads reported that they recognized their interaction partners. These participants and their partners (*N*_*dyad*_ = 6, *N*_*participant*_ = 12) were also removed from data analyses, resulting in a final sample size of 248 participants (*N*_*dyad*_ = 124).

#### Interpersonal attraction

**Figure 8:**
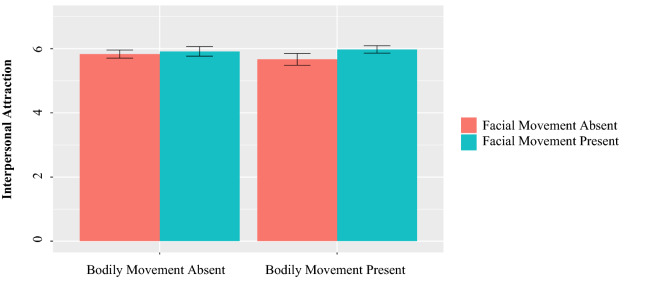
Mean interpersonal attraction by condition.

There was a significant main effect of facial movements on interpersonal attraction (Fig. 8), such that dyads that were able to see their partner’s facial movements mapped on their avatars felt higher levels of interpersonal attraction than those that were unable to see these facial movements (*b* = 0.09, *p* = 0.02, *d* = 0.30). In contrast, the availability of bodily movements did not significantly influence interpersonal attraction (*b* = − 0.02, *p* = 0.57). The interaction effect between facial and bodily movements was also non-significant (*b* = 0.05, *p* = 0.17).

#### Affective valence

**Figure 9:**
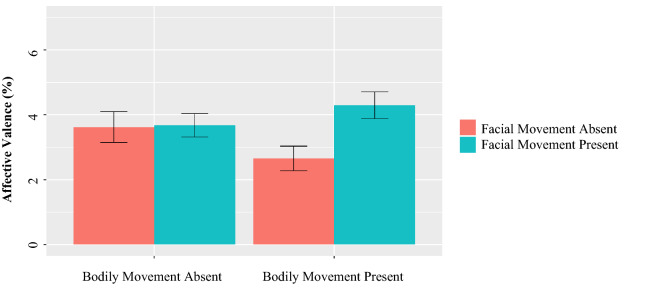
Mean affective valence by condition.

There was a significant interaction between facial and bodily movements (*b* = 0.46, *p* = 0.03, Fig. 9). Simple effects tests showed that while dyads that could see their partner’s facial movements described their experience more positively, this was only true when their partner’s bodily movements were also visible (*b* = 0.84, *p* = 0.01, *d* = 0.50); in contrast, the positive effect of facial movement on affective valence was non-significant when bodily movements were not visible (*b* = − 0.07, *p* = 0.80). These results suggest that dyads only described their experiences most positively when they were able to see both their partner’s bodily movements and their facial movements, lending partial support to studies that showed a preference for representation consistency^86^.

#### Impression accuracy

**Figure 10:**
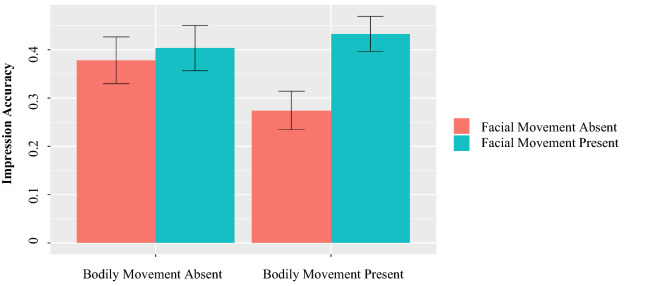
Mean impression accuracy by condition.

Impression accuracy was significantly and positively influenced by the availability of facial movements (*b* = 0.06, *p* = 0.02, *d* = 0.34, Fig. 10). In contrast, being able to see one’s partner’s bodily movements did not influence impression accuracy (*b* = − 0.01, *p* = 0.60). The interaction between facial and bodily movements was also non-significant (*b* = 0.03, *p* = 0.27).

#### Social presence

Neither the availability of facial movements (*b* = 0.04, *p* = 0.29) nor the availability of bodily movements (*b* = 0.04, *p* = 0.31) had a significant effect on social presence. The interaction effect between facial and bodily movements was also non-significant (*b* = 0.06, *p* = 0.16).

#### Extent of bodily movement

Dyads who were able to see their partner’s bodily movements being mapped on to their partner’s avatars moved their body more (*b* = 0.02, *p* < 0.0001), although this main effect was qualified by a significant interaction effect (*b* = 0.01, *p* = 0.048). Simple effects tests showed that dyads who could see their partner’s bodily movements moved more when their partner’s facial movements were also visible (*b* = 0.04, *p* < 0.001, *d* = 0.89); this effect of bodily movement was only marginally significant when their partner’s facial movements were not visible (*b* = 0.01, *p* = 0.09).

#### Extent of facial movement

In contrast to bodily movements, the visibility of one’s partner’s facial movements did not influence the extent to which dyads moved their faces (*b* = − 0.0004, *p* = 0.79). Neither the main effect of bodily movements (*b* = 0.001, *p* = 0.60) nor the interaction effect between facial and bodily movements were significant (*b* = 0.002, *p* = 0.18).

#### Nonverbal synchrony

The visibility of facial movements positively predicted synchrony in facial movements (*b* = 0.01, *p* < 0.001), while the presence of bodily movement did not predict facial synchrony (*b* = − 0.0002, *p* = 0.95); the interaction term between face and body was also non-significant (*b* = 0.00004, *p* = 0.99). Gender significantly predicted facial synchrony, such that females displayed higher facial synchrony than males (*b* = 0.02, *p* < 0.001).

Dyads that were able to see their partner’s bodily movements exhibited marginally higher levels of bodily synchrony compared to those that were unable to see each other (*b* = 0.002, *p* = 0.09, *d* = 0.28). Neither the presence of facial movement nor gender significantly predicted synchrony in bodily movement (both *p*s > 0.10). The interaction term was also non-significant (*b* = − 0.001, *p* = 0.62).

**Figure 11:**
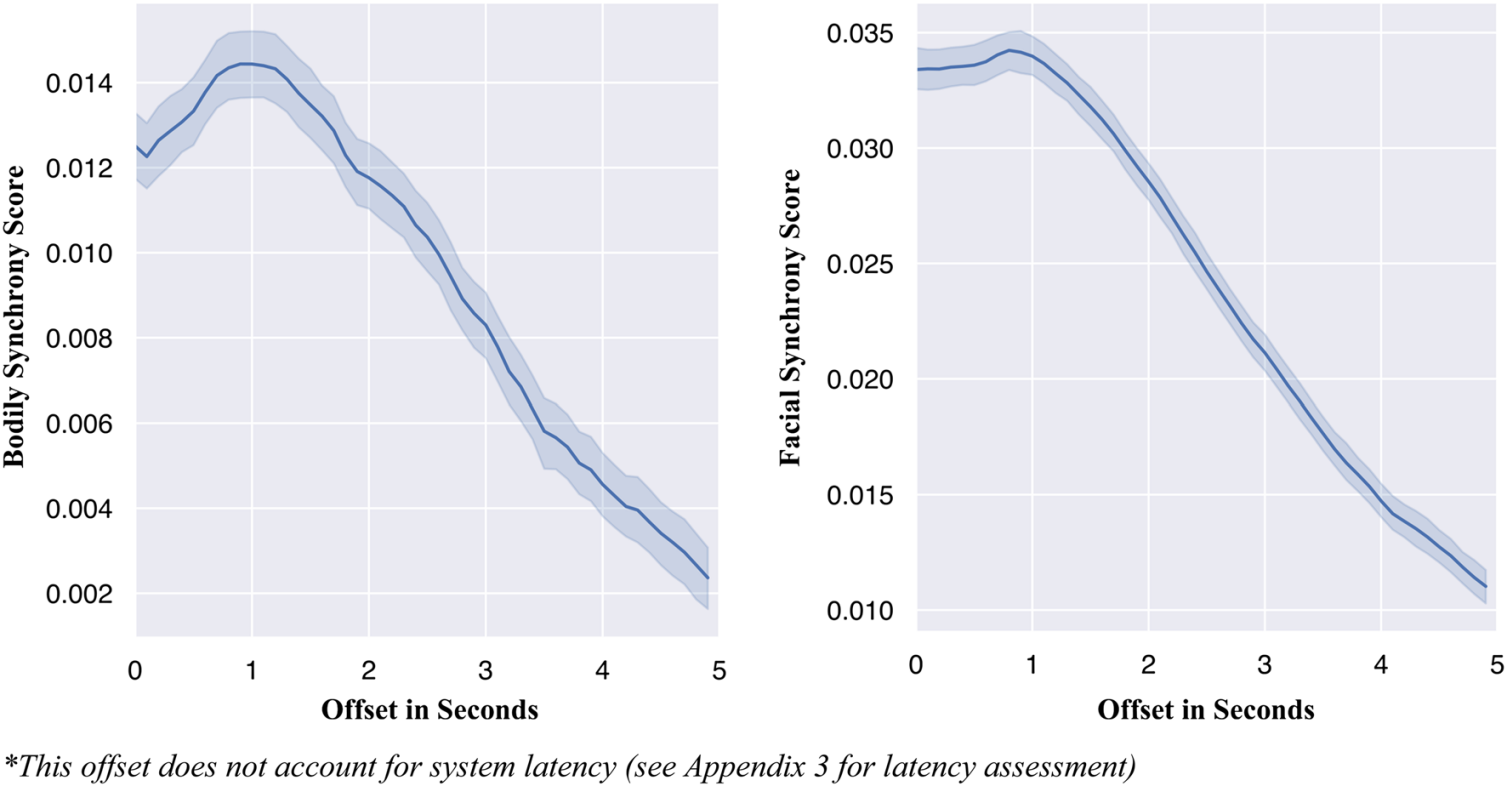
Averaged correlations of bodily (left) and facial (right) movements: represents changes in synchrony scores based on offset interval*.

To assess the robustness of the synchrony measure, we explored synchrony patterns across different time lags (Fig. 11) and found that synchrony scores decrease as the time lag increases for both facial and bodily synchrony, which suggests that the scores are representative of true synchrony^42^. That is, as the time lag between the two streams of each participant’s nonverbal data increases, the synchrony score approaches closer to zero, which is the expected pattern, given that nonverbal synchrony is defined as the “temporal co-occurrence of actions”^87^. T-tests also showed that both synchrony scores were significantly different from zero (Bodily Synchrony: *t*(245) = 14.72, *p* < 0.001; Facial Synchrony: *t*(244) = 14.66, *p* < 0.001), with a large effect size (Cohen’s *d* = 0.939 and Cohen’s *d* = 0.937 for bodily synchrony and facial synchrony, respectively).

### Movement data and interpersonal attraction

**Figure 12:**
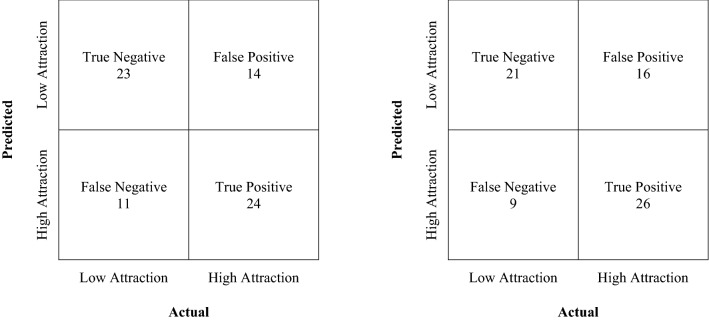
Confusion table for random forest model (left) and multi-layer perceptron model (right).

Both classifiers were able to predict interpersonal attraction at an accuracy rate higher than chance, suggesting that automatically detected nonverbal cues can be used to infer interpersonal attitudes. After tuning the hyperparameters (Table 5) based on the cross-validation performance of the training set, the random forest model achieved a cross-validation accuracy of 67.33% (*SD* = 8.28%) and a test accuracy of 65.28%; the MLP model achieved a cross-validation accuracy of 68.67% (*SD* = 5.63%) and a test accuracy of 65.28% (majority class baseline: 51.39%). Confusion tables that depict sensitivity and specificity assessments for the two models are in Fig. 12.

**Table 5:** Hyperparameters and values.

Hyper-parameter/classifier	Random forest	MLP
activation	–	logistic
α	–	0.03
batch_size	–	auto
β_1_	–	0.9
β_2_	–	0.999
bootstrap	True	–
criterion	entropy	–
early_stopping	–	False
ε	–	110–8
hidden_layer_sizes	–	(50)
learning_rate	–	constant
learning_rate_init	–	0.001
max_depth	10	–
max_features	20	–
max_iter	–	200
max_leaf_nodes	None	–
min_impurity_decrease	0	–
min_impurity_split	None	–
min_samples_leaf	7	–
min_samples_split	2	–
min_weight_fraction_split	0	–
momentum	–	0.9
n_iter_no_change	–	10
n_*esterovs_momentum*_	–	True
n_*estimators*_	500	–
n_*jobs*_	None	–
Power_*t*_	–	0.5
random_state	30	30
shuffle	–	True
solver	–	adam
tol	–	110–4
oob_score	False	–
validation_fraction	–	0.1

## Discussion

The present study aimed to understand the relative and joint influence of facial and bodily cues on communication outcomes. Contrary to hypotheses based on behavioral realism, the inclusion of bodily gestures alone did not have a significant main effect on interpersonal attraction, social presence, affective valence, and impression formation. Additionally, when facial cues were not available, LIWC data suggested that participants felt more positively when bodily gestures were *not* available, compared to when they were. These results are in line with studies that did not find support for the conjecture that avatar movement would increase social presence or improve interpersonal outcomes^30,31^. At the same time, they appear to contradict previous research and theories suggesting that additional social cues and/or social realism lead to higher levels of social presence and more positive communication outcomes^21,22,88,89^. In contrast to the null effect of including bodily gestures, the present study found evidence that the presence of facial expressions can moderately improve communication outcomes across multiple dimensions, including interpersonal attraction, affective valence, and impression accuracy.

The null main effect of bodily gestures on interpersonal outcomes may, at least in part, be explained by the following mechanisms. First, participants may have been able to compensate for the lack of bodily cues with the other cues at their disposition (e.g., verbal cues). This explanation is in line with previous CMC theories (e.g., Social Information Processing Theory^32^), which found that increased interaction time allows interactants to overcome the lack of nonverbal cues available. At the same time, the positive interpersonal effects of facial cues suggest that, at minimum, facial cues offered a unique value to participants within the current avatar-mediated context that bodily cues did not.

Second, bodily movements may have been less relevant than facial movements and speech within the context of the present study. Although we adopted a visual and semantic referential task to encourage both nonverbal and verbal communication, the presence (or absence) of bodily movements was not an integral part of completing the tasks. In addition, because the participants were not immersed in the same virtual space (i.e., communicated in separate rooms through a screen), it is possible that they lacked the common ground to effectively communicate via gestures. Considering that the interaction context heavily influences the communicational value of gestures^90,91^ the inclusion of gestures may have yielded more positive outcomes if participants had been communicating within a context where gestures carried higher semantic and practical value.

In addition to the specific requirements of the tasks performed by the participants, the experimental setup itself may have encouraged participants to focus on the avatar’s face, rather than its body. As depicted in Fig. 2, participants interacted with an avatar whose representation was limited to the upper body. This was an intentional choice primarily due to the limitations of the Kinect in tracking lower body joints. However, it is possible that the lack of ‘full body representation’ led to a cognitive bias favoring the face. Taken together with the results of the present study, it appears that upper body gestures within separate (‘non-shared’) virtual spaces may be relatively less important for dyadic interactions.

A final explanation for the null—and in some cases, negative—impact of bodily movements, however, may be that the technical limitations of the systems led to poor body tracking. While plausible, the fact that participants who were able to see their partner’s facial expressions and bodily movements described their experience the most positively suggests that, at the very least, technical limitations were not uniquely responsible for the negative impact of bodily movements on affective valence. That is, even when considering the technical limitations, having access to bodily gestures had a positive impact on affective valence when they were coupled with facial expressions. This is consistent with Aviezer and colleagues^12^ who argue that facial and bodily cues are processed as a unit rather than independently.

While the accuracy rate of the machine learning model was weak (approximately 65%), it is important to note that interpersonal attitudes are difficult for even human judges to predict. For example, judges who viewed videotaped interactions between two individuals were able to rate interpersonal rapport at an accuracy rate that was higher than chance, but the effect size was fairly small^92^ (i.e., *r* = 24). In addition, it is important to note that previous studies showed inconclusive evidence that machine learning could be applied to consistently predict interpersonal attitudes for a non-selective data set. For instance, the accuracy rate of previous studies^42,51^ were at chance level when the classifier was applied to the entire dataset, and were above chance only when data set selection was exclusive (i.e., increasingly removing interaction pairs that scored closer to the median). Similarly, the validation accuracy rate for Jacques and colleagues^36^ was close to chance level (approximately 5% higher than baseline), which is a relatively large difference from the testing set accuracy (approximately 20% higher than baseline), a limitation which is also noted by the authors. Albeit low, the present study shows validation and test accuracy rates that are both approximately 15% higher than the baseline, offering stronger evidence that machine learning can be applied to the prediction of more complex interpersonal outcomes.

Investigating which cues most strongly influence avatar-mediated interactions can help researchers isolate the cues that people rely on to form affective and cognitive judgments about others and communication experiences using an inductive process. While the majority of extant studies have used deductive processes to test whether specific nonverbal cues would affect user perceptions of virtual interactions^30,93,94^, only a select number of studies have jointly relied on inductive processes (e.g., machine learning) to isolate cues that contribute most strongly to interpersonal outcomes^36^. Machine learning can help identify significant nonverbal cues for interpersonal outcomes through feature selection processes and model comparisons. Identifying and testing these cues can help inform theories of person perception and impression formation. Recent advancements in facial and motion tracking technology and computing power render this bottom-up approach particularly attractive for nonverbal theory development.

From a practical standpoint, identifying nonverbal cues with the strongest social influence can help VR designers and engineers prioritize features that should be available within virtual environments. Given the amount of resources that are being invested into developing social VR platforms, understanding where to focus development efforts can aid in allocating resources more effectively. For instance, the present study suggests that facial animations are critical for positive avatar-mediated interactions, especially when there are bodily movements. As such, the development of avatars that are able to both express realistic facial expressions and credibly transition between expressions coupled with technologies that can accurately track the user’s facial expressions in real time could improve interpersonal outcomes and improve human–machine interactions. Within the context of immersive VR, however, most of the tracking technology has thus far focused on body tracking (e.g., Oculus Touch, HTC Vive Lighthouse). This bias is likely due to the fact that most of these systems rely on bodily nonverbal behavior as input to render the virtual environment appropriately. Additionally, the use of head-mounted displays makes it challenging to track facial expressions. The current findings offer some evidence that social VR platforms, immersive or not, may benefit from investing in technologies that can capture (or infer) and map facial expressions within avatar-mediated environments.

This investigation employed a novel technical set up that allowed for the activation and deactivation of specific nonverbal channels to study their individual and joint effects on interpersonal outcomes. Our setup differentiates itself from prominent social VR applications, which are generally limited to body tracking. While a small number of applications do support face tracking, these have remained relatively costly solutions that aren’t widely available. We demonstrate a solution capable of tracking both the face and body by combining ubiquitously available consumer electronics.

Outside the study of avatar-mediated environments, this setup could be adapted by nonverbal communication researchers to further understand the impact of specific nonverbal channels during FtF interaction and help address methodological challenges associated with manually coding nonverbal behavior or reduced ecological validity (e.g., having to block out specific body parts^19^). Additionally, with the increasing availability of large data sets of automatically detected nonverbal behavior, inductive processes can be leveraged to produce bottom-up algorithms^42^ that can help identify nonverbal patterns during specific interactions that cannot be perceived by the human eye.

## Limitations

It is important to note the limitations associated with the present study. First, the technical setup of the present study focused on the tracking and rendering of nonverbal cues, but did not account for dimensions such as stereoscopic viewing or perspective dependency. This limits the generalizability of our findings to contexts wherein different VR technologies are utilized. Future studies would benefit from exploring the interplay between different technological affordances and the availability of nonverbal cues.

Second, our focus was limited to two nonverbal channels: body and face. As such, we were unable to explore the effects of additional nonverbal cues such as tone or intonation. While this is beyond the scope of the present study, future research should explore the impact of these cues along with facial and nonverbal behavior to better understand the effects of various nonverbal channels on interaction outcomes.

Another limitation of the study lies in the relatively specific interaction context wherein participants were asked to collaborate on one visual and one semantic referential task. This decision was made primarily to avoid ceiling effects on impression formation^58^ and to control for the variance in communication content (e.g., extent of self-disclosure) that can influence interpersonal outcomes. However, it is likely that the task-centered nature of the interaction context restricted the social and affective aspects of the interaction, which may have limited the role of nonverbal communication. Furthermore, due to the collaborative nature of the task, participants may have been more prone to display favorable nonverbal cues. The specificity of the current context also reduces the generalizability of the current findings, as everyday interactions are characterized by a combination of both task-oriented and social content^95,96^. Future studies should employ different interaction contexts to understand potential boundary conditions.

Additionally, while we simultaneously varied facial and bodily cues for the visual referential task (see “Methods”), it is possible that participants found this task to be biased toward facial expressions as they resembled emojis, rendering facial expressions more salient than bodily cues. Follow-up studies should thus sample different tasks to account for stimuli effects^97^.

Finally, the technical limitations associated with markerless tracking need to be addressed. While the present study used two of the most precise motion tracking systems that are currently available, there were still limitations in terms of the range of movements that the systems could track. For instance, participants needed to stay within a specific distance from the facial tracking camera in order to ensure smooth tracking (see “Methods”) and touching the face or turning the head completely away from the camera resulted in tracking errors. In addition, while our latency was within the established range for video-based communication (“Appendix 4”), it is unlikely that our system was able to reliably capture and render micro-expressions.

The Kinect was also limited in its tracking when there was an overlap between joints (e.g., when the participant crossed his or her arms) and for certain rotation angles. Because this tracking data was used to animate the avatars, it is probable that these technical limitations led to instances wherein the movements of the avatar appeared unnatural. While this was an inevitable limitation given the current state of the technology, more studies should be conducted as motion tracking technology continues to advance.

## Conclusion

The present study found that people who are able to see their partner’s facial cues mapped on their avatars like their partners more and form more accurate impressions in terms of personality. Contrary to hypotheses, the availability of bodily cues alone did not improve communication outcomes. In addition, we found that machine learning classifiers trained with automatically tracked nonverbal data could predict interpersonal attraction at an accuracy rate that was approximately 15% higher than chance. These findings provide new insights on the individual and joint interaction of two nonverbal channels in avatar-mediated virtual environments and expand on previous research suggesting that the automatic detection of nonverbal cues can be used to predict emotional states. This is particularly prescient as technology makes it increasingly easy to automatically detect and quantify nonverbal behavior.

## Figures and Tables

**Figure 13:**
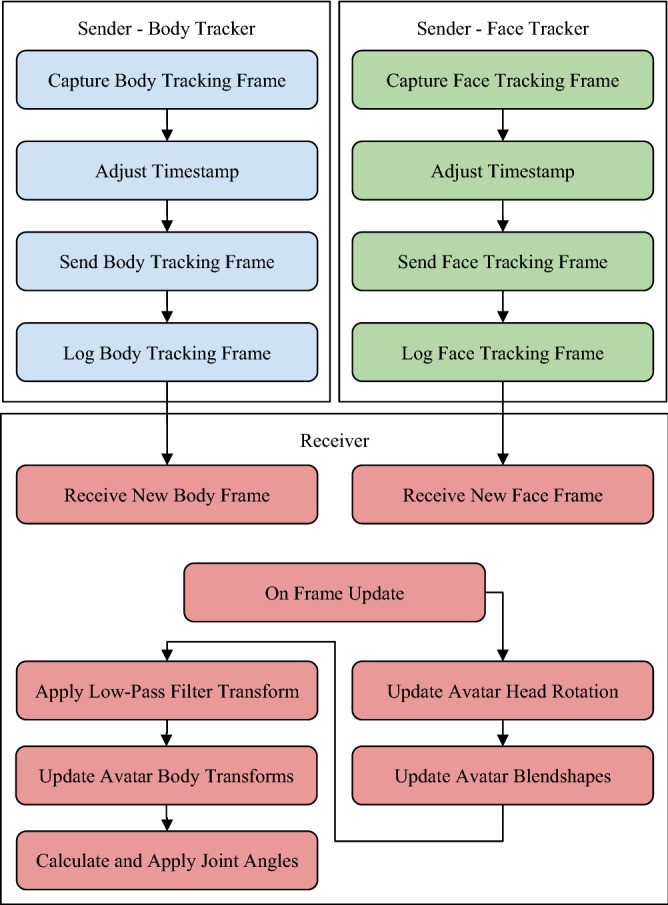
Sequence chart of how body and face tracking data were processed.

**Figure 14:**
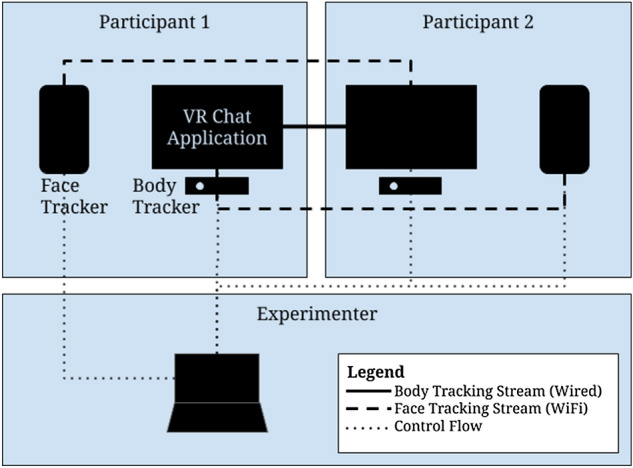
Network diagram of the connections between the devices.

## Data Availability

The datasets generated during and/or analyzed during the current study are available from the corresponding author on reasonable request.

## Appendix 1: Facial movements tracked by Apple iPhone^55^

**Table:**

Apple blendshapes	Description
browDownLeft	Downward movement of outer portion of left eyebrow
browDownRight	Downward movement of outer portion of right eyebrow
browInnerUp	Upward movement of inner portion of left and right eyebrows
browOuterUpLeft	Upward movement of outer portion of left eyebrow
browOuterUpRight	Upward movement of outer portion of right eyebrow
cheekPuff	Outward movement of both cheeks
cheekSquintLeft	Upward movement of cheek around and below the left eye
cheekSquintRight	Upward movement of cheek around and below the right eye
eyeBlinkLeft	Closure of the eyelid over the left eye
eyeBlinkRight	Closure of the eyelid over the right eye
eyeLookDownLeft	Movement of the left eyelid consistent with a downward gaze
eyeLookDownRight	Movement of the right eyelid consistent with a downward gaze
eyeLookInLeft	Movement of the left eyelid consistent with an inward gaze
eyeLookInRight	Movement of the right eyelid consistent with an inward gaze
eyeLookOutLeft	Movement of the left eyelid consistent with an outward gaze
eyeLookOutRight	Movement of the right eyelid consistent with an outward gaze
eyeLookUpLeft	Movement of the left eyelid consistent with an upward gaze
eyeLookUpRight	Movement of the right eyelid consistent with an upward gaze
eyeSquintLeft	Contraction of the face around the left eye
eyeSquintRight	Contraction of the face around the right eye
eyeWideLeft	Widening of the eyelid around the left eye
eyeWideRight	Widening of the eyelid around the right eye
jawForward	Forward movement of the lower jaw
jawLeft	Leftward movement of the lower jaw
jawOpen	Opening of the lower jaw
jawRight	Rightward movement of the lower jaw
mouthClose	Closure of the lips independent of jaw position
mouthDimpleLeft	Backward movement of the left corner of the mouth
mouthDimpleRight	Backward movement of the right corner of the mouth
mouthFrownLeft	Downward movement of the left corner of the mouth
mouthFrownRight	Downward movement of the right corner of the mouth
mouthFunnel	Contraction of both lips into an open shape
mouthLeft	Leftward movement of both lips together
mouthRight	Rightward movement of both lips together
mouthLowerDownLeft	Downward movement of the lower lip on the left side
mouthLowerDownRight	Downward movement of the lower lip on the right side
mouthPressLeft	Upward compression of the lower lip on the left side
mouthPressRight	Upward compression of the lower lip on the right side
mouthPucker	Contraction and compression of both closed lips
mouthRollLower	Movement of the lower lip toward the inside of the mouth
mouthRollUpper	Movement of the upper lip toward the inside of the mouth
mouthShrugLower	Outward movement of the lower lip
mouthShrugUpper	Outward movement of the upper lip
mouthSmileLeft	Upward movement of the left corner of the mouth
mouthSmileRight	Upward movement of the right corner of the mouth
mouthStretchLeft	Leftward movement of the left corner of the mouth
mouthStretchRight	Rightward movement of the right corner of the mouth
mouthUpperUpLeft	Upward movement of the upper lip on the left side
mouthUpperUpRight	Upward movement of the upper lip on the right side
noseSneerLeft	Raising of the left side of the nose around the nostril
noseSneerRight	Raising of the right side of the nose around the nostril
tongueOut	Extension of the tongue

## VR chat application (face and body tracker)

The face tracker was implemented as an iOS application running on an iPhone XS. Apple’s ARKit 2.0 SDK, which is built into the iPhone XS, was used to extract tracking status, continuous facial features, and rotation data of the eyes and head. All facial features as well as eye rotation were mapped to the corresponding blendshapes of the avatar head model.

While both the iPhone and Kinect can track head rotation, we found the iPhone data to be more reliable. As such, the head rotation provided by the iPhone XS was used as the primary input data for avatar animation; the head rotation data provided by the Kinect was used as a fallback for instances wherein the iPhone XS failed to track the participant. The face model used for the avatar in the study was Mateo 3D model by Faceshift, licensed under Creative Commons Attribution 3.0. For the female avatar, the same model was used, but the hair was created separately by our lab.

The VR Chat Application was implemented as a Unity application running on a Windows PC and includes the body tracker, and experiment overlay. It includes the body tracker which uses the Kinect for Windows SDK 2.0 and the corresponding Unity plugin. The body model used in the study was AJ 3D Model by Mixamo (Adobe Systems). All Kinect joints from spine base and upward were mapped to the model depicted in Fig. 2. While the Kinect reports joint rotation, we found that this performed poorly on arm joints and therefore rotation data were only used for spine joints. Arm, hand and shoulder joint rotation was inferred using inverse kinematics. A detailed list of the software programs used in the current study is as follows:

**Table:**

Software	Version
Unity	2018.1.6f1
Kinect for Windows SDK	2.0.1410.19000
iOS on iPhone XS	12.1
protobuf	3.1.0
python	3.7.4
numpy	1.16.4
pandas	0.24.2
sklearn	0.21.3

## Control panel

The control panel was implemented as a Unity application running on a Windows PC. It allows the Experimenter to monitor the tracking and connection status of all trackers. It was also used to configure, calibrate, start, record responses, pause, resume and conclude the experiment. A diagram of how the body and face tracking data were processed can be seen in Fig. 13 and a network diagram of the connections between the devices is available in Fig. 14. 

## Appendix 3: Latency assessment for experimental setting

System latency was computed based on the latency of the subsystems. The latency of each individual component is listed in the table below. ARKit provides a capture timestamp, which was used to measure capture delay throughout the study. As the Kinect lacks this feature, we relied on previous research by Waltemate and colleagues^98^. We observed network latency and variance for the face trackers that we connected via wireless network. In order to achieve the required time synchronization between trackers, we timestamped a message when captured, sent, received and rendered, and use a time synchronization approach^99^ to calculate time offset and network delay. The rolling average and standard deviation of the calculated latencies were logged every second. We calculate the render delay as the difference between the time the data is received and the time when Unity completed rendering the frame.

While 100 ms is established as a safe latency that ensures user satisfaction in video conferencing^100^, a more recent study^101^ suggests that latencies as high as 500 ms do not have a significant negative impact on likeability and naturalness. Of note, there were no complaints regarding system performance during the pilot study with 40 participants, which is expected as our total latency was within the established target range. In addition to the approach taken in the present study, future studies may also benefit from conducting a video-based assessment in order to determine motion-to-photon latencies.

**Table:**

	Body tracking	Face tracking
Sensor/capture delay	98.8 ± 19.2 ms	84.8 ± 9.0 ms
Network stream latency	< 1 ms	8.5 ± 33.9 ms
Render delay	30.4 ± 10.9 ms	
Display response delay	8 ms*	
Total	138.2 ± 22.1 ms	131.7 ± 36.7 ms

*As reported by the display manufacturer.

## Social presence^74,75^

How strongly do you agree or disagree with the following statements about your partner?

**Table:**

1	2	3	4	5	6	7
Strongly disagree	Disagree	Somewhat disagree	Neither agree nor disagree	Somewhat agree	Agree	Strongly agree

1. I felt that my partner was present.
2. I felt that my partner was aware of my presence.
3. I paid close attention to my partner.
4. My partner paid close attention to me.
5. I was influenced by my partner's emotions.
6. My partner was influenced by my emotions.
7. My thoughts were clear to my partner.
8. My partner's thoughts were clear to me.

## Interpersonal attraction^62, 63^

### Task attraction

How strongly do you agree or disagree with the following statements about your experience?

**Table:**

1	2	3	4	5	6	7
Strongly disagree	Disagree	Somewhat disagree	Neither agree nor disagree	Somewhat agree	Agree	Strongly agree

1. I enjoyed completing the tasks with my partner.
2. I had fun completing the tasks with my partner.
3. I would like to interact with my partner again.
4. It was interesting to complete the tasks with my partner.

### Social attraction

How strongly do you agree or disagree with the following statements about your partner?

**Table:**

1	2	3	4	5	6	7
Strongly disagree	Disagree	Somewhat disagree	Neither agree nor disagree	Somewhat agree	Agree	Strongly agree

1. I like my partner.
2. I would get along well with my partner.
3. I would enjoy a casual conversation with my partner.
4. My partner is friendly.

## Impression Accuracy (Short 15-item big five inventory; BFI-S^70,71^)

### BFI-S observer version

You will now see a number of statements, each of which starts with, "I see MY PARTNER as someone who…". For each statement, indicate how much you agree or disagree with this. If you are unable to make a judgment, select "Cannot make judgment".

**Table:**

1	2	3	4	5	6	7	N/A
Strongly disagree	Disagree	Somewhat disagree	Neither agree nor disagree	Somewhat agree	Agree	Strongly agree	Cannot make judgment

### BFI-S self version

You will now see a number of statements, each of which starts with, "I see MYSELF as someone who…". For each statement, indicate how much you agree or disagree with this.

**Table:**

1	2	3	4	5	6	7
Strongly disagree	Disagree	Somewhat disagree	Neither agree nor disagree	Somewhat agree	Agree	Strongly agree

**Table:**

Trait	Items
Openness to experience	comes up with new ideas
	values artistic experiences
	has an active imagination
Conscientiousness	does a thorough job
	tends to be lazy
	does things efficiently
Extroversion	is talkative
	is outgoing
	is reserved
Agreeableness	is sometimes rude to others
	has a forgiving nature
	is kind
Neuroticism	worries a lot
	gets nervous easily
	remains calm in tense situations
